# Risk of nonmelanoma skin cancer in patients with psoriatic arthritis and according to bDMARD treatment—a cohort study from the Nordic Arthritis Research Collaboration

**DOI:** 10.1016/j.ero.2026.03.009

**Published:** 2026-04-09

**Authors:** Rasmus Westermann, Bénédicte Delcoigne, René Lindholm Cordtz, Sella Aarrestad Provan, Joe Sexton, Dan Nordström, Pia Isomäki, Johan Askling, Lene Wohlfahrt Dreyer, Karin Hellgren

**Affiliations:** 1 Center of Rheumatic Research Aalborg (CERRA), Department of Rheumatology, Aalborg University Hospital, Aalborg University, Aalborg, Denmark; 2 Clinical Cancer Research Center, Aalborg University Hospital, Aalborg, Denmark; 3 Clinical Epidemiology Division, Department of Medicine Solna, Karolinska Institutet, Stockholm, Sweden; 4 Division of Rheumatology and Research, Diakonhjemmet Hospital, Oslo, Norway; 5 Department of Medicine and Rheumatology, Helsinki University Central Hospital, Helsinki, Finland; 6 Centre for Rheumatology, Tampere University Hospital and Faculty of Medicine and Health Technology, Tampere University, Tampere, Finland; 7 The DANBIO registry, Glostrup, Denmark; 8 Academic Specialist Center, Stockholm Health Services, Stockholm, Sweden

## Abstract

**Objectives:**

To investigate if psoriatic arthritis (PsA) itself and if biological disease-modifying antirheumatic drugs (bDMARDs) used in PsA are associated with increased risks of nonmelanoma skin cancer (NMSC).

**Methods:**

From nationwide health and clinical rheumatology registers in 4 Nordic countries, we identified patients with PsA who between 2010 and 2021 started a: (i) tumour necrosis factor inhibitor (TNFi), or (ii) non-TNFi bDMARD, or were (iii) b/targeted synthetic (ts)DMARD naïve. For Sweden and Denmark, we identified incident patients with PsA (2010-2021) and corresponding general population (GP) comparator subjects. Using ever-treated follow-up, incidence rates and adjusted hazards ratios (aHRs) with 95% CIs were calculated for NMSC overall, basal cell carcinoma (BCC), and squamous cell carcinoma (SCC), respectively.

**Results:**

We identified 619 NMSCs among incident patients with PsA (n = 23,553). We observed 221 NMSCs in TNFi-treated (n = 9999), 34 NMSCs in non-TNFi bDMARD-treated (n = 2200), and 433 NMSCs in b/tsDMARD-naïve (n = 19,089) PsA. The aHR of NMSC for incident PsA vs GP was 1.20 (1.11-1.31). Pooled aHRs for NMSC overall were 1.24 (1.03-1.50) with TNFi and 1.11 (0.75-1.63) with non-TNFi bDMARDs vs b/tsDMARD-naïve PsA. Corresponding aHRs of BCC were 1.21 (0.93-1.58) for TNFi and 0.85 (0.42-1.69) for non-TNFi bDMARDs, whereas aHRs for SCC were 1.46 (0.84-2.56) and 2.37 (0.95-5.90).

**Conclusions:**

Increased risks of NMSC (SCC in particular) were found in patients with PsA compared with the GP. Compared with b/tsDMARD-naïve PsA, TNFi was associated with an increased risk of NMSC overall, whereas a nonsignificantly increased risk of SCC was seen for non-TNFi bDMARDs. However, causality between bDMARD use and NMSC in PsA remains uncertain.

**WHAT IS ALREADY KNOWN ON THIS TOPIC**

• Some studies suggest an increased risk of nonmelanoma skin cancer (NMSC) in patients with psoriatic arthritis (PsA), yet evidence is limited regarding the role of biological disease-modifying antirheumatic drug (bDMARD) on NMSC risk in PsA.

**WHAT THIS STUDY ADDS**

• We observed increased risks of NMSC, both basal cell carcinoma (BCC) and squamous cell carcinoma (SCC), in incident PsA compared with matched comparators from the general population.
• Treatment with tumour necrosis factor inhibitor (TNFi) was associated with an increase in NMSC risk (with numeric increases for both BCC and SCC) compared with b/targeted synthetic (ts)DMARD-naïve PsA patients, corresponding to 1020 patients requiring 1 year of treatment before observing 1 excess NMSC.
• Treatment with non-TNFi bDMARDs was neither associated with any overall increased risk of NMSC nor of BCC. A more than doubled incidence of SCC was seen compared with b/tsDMARD-naïve PsA patients, which corresponded to 694 patients requiring 1 year of treatment before observing 1 excess SCC.

**HOW THIS STUDY MIGHT AFFECT RESEARCH, PRACTICE OR POLICY**

• Patients with PsA experience increased risks of NMSC. Irrespective of causality, the risk is higher among those treated with bDMARDs. Contextualisation on an absolute scale is warranted before implementing cautionary strategies into clinical practice.

Alt-text: Unlabelled box dummy alt text

## INTRODUCTION

Psoriatic arthritis (PsA) is a chronic inflammatory disease featuring joint inflammation, enthesitis, axial manifestations, and cutaneous involvement [1,2]. PsA is associated with considerable comorbidity, potentially including nonmelanoma skin cancer (NMSC) [[3], [4], [5], [6]]. Worldwide NMSC comprises about one-third of all diagnosed malignancies each year and consists primarily of 2 subtypes: basal cell carcinoma (BCC) and squamous cell carcinoma (SCC) [[7], [8], [9]]. Exposure to ultraviolet (UV) radiation is the most established risk factor for NMSC, whereas other risk factors include fair skin complexion, immune incompetence due to organ transplantation, HIV, or use of immunosuppressive drugs [[7], [8], [9]]. Available literature suggests a 50% to 100% increase in the risk of NMSC in patients with cutaneous psoriasis (PsO) compared with the general population (GP), with higher risks of SCC than of BCC [[3], [4], [5],^10^], particularly among those exposed to psoralen UV-A treatment [11,12]. Corresponding studies in patients with PsA are sparse, but with indications of increased NMSC risks of a magnitude similar to that of PsO [[3], [4], [5], [6]].

The pathological mechanisms linking PsO/PsA to NMSC development are not fully understood [13]. The beneficial effects of UV light on psoriatic skin might—via artificial phototherapy and a heightened sun-seeking behaviour—partly explain the excess NMSC risk in this patient population. The chronic inflammatory characteristics of the underlying condition with regard to disease duration and severity might also be of importance, as may local and/or systemic treatment against PsO/PsA. Furthermore, patients with PsO/PsA more often undergo skin examinations by clinicians than the GP, in which case NMSC may be diagnosed more frequently. A meta-analysis from 2019 found that the risks of BCC and SCC were considerably higher among patients with severe PsO than for patients with PsO overall [14]. Some studies on PsO/PsA have also indicated higher risks of NMSC following conventional synthetic disease-modifying antirheumatic drugs (csDMARDs) such as methotrexate (MTX) and following targeted synthetic (ts)DMARDs [[15], [16], [17], [18], [19], [20]]. Today’s mainstay therapy for PsA includes csDMARDs, biological (b)DMARDs, and tsDMARDs. Although tumour necrosis factor inhibitors (TNFis) are the most commonly used type of bDMARD in PsA, non-TNFi bDMARDs acting via interleukin (IL) inhibition (IL-17A, IL-17A/F, IL-12/23, or IL-23) are increasingly used [21]. Yet, there are few studies of NMSC risk with bDMARD use in PsA [5,18,[22], [23], [24], [25]]. Corresponding data on NMSC risk with TNFi treatment in PsO are inconsistent: 1 meta-analysis did not observe any increased risk of NMSC with TNFi [4], whereas other studies have suggested increased risks, especially for SCC [5,18,[26], [27], [28]]. The limited available data for PsA also contains signals of an increased NMSC risk following treatment with TNFi, but most studies are based on GP comparisons, which complicates TNFi-specific inferences if the underlying PsO/PsA in itself increase NMSC risk [5,22,23]. For non-TNFi bDMARDs in PsA, evidence on the risk of NMSC is scarce [18,24].

For all the above reasons, we set out to investigate the underlying risk of NMSC overall and by the 2 main subtypes, SCC and BCC, in incident patients with PsA compared with the GP, and to assess the risk with TNFi- and non-TNFi bDMARD use in PsA.

## METHODS

### Study design and setting

We performed an observational cohort study of NMSC risk in patients with PsA from 4 Nordic countries between January 1, 2010 to December 31, 2021 based on prospectively collected data from nationwide health and clinical rheumatology registers (CRRs). In all Nordic countries, health care systems are tax-funded, and individual-level information across registers is available within each country by linkage via the unique personal identification numbers assigned to all residents.

### Data sources

Patients with PsA and their bDMARD treatment status were identified from the 4 Nordic CRRs: DANBIO (Denmark), NOR-DMARD (Norway), ROB-FIN (Finland), and SRQ (Sweden) [29]. All CRRs used have been described in detail elsewhere [29,30]. In the CRRs, the diagnosis of PsA is set by the treating rheumatologist. In addition, the CRRs contain longitudinal information on DMARD treatments including start and stop dates and PsA disease-related information such as duration and activity. Information on comorbidities, education, country of birth, emigration, and vital status was collected via linkage to National Patient Registers (NPRs) as well as from various population registers [[31], [32], [33], [34], [35], [36], [37], [38], [39]]. For Sweden and Denmark, a cohort of incident patients with PsA and matched GP comparator subjects was identified from the Swedish and Danish NPRs and population registers, respectively. For all countries, we captured information on NMSCs from National Cancer Registers containing information on date and type of cancer according to the International Classification of Diseases (ICD-10) and Systematized Nomenclature of Medicine (SNOMED) coding [40]. Reporting to the Nordic National Cancer Registers is mandatory, and semiautomated, resulting in high coverage [[41], [42], [43], [44]]. The study period for both inclusion of patients with PsA and follow-up for NMSC outcomes was from January 1, 2010, to the end of 2021 for Sweden and Denmark, the end of 2018 for Finland, and the end of 2019 for Norway.

### Study population and exposure

For this study, we defined 4 potentially overlapping cohorts of patients with PsA according to bDMARD treatment status and registry identification (CRR vs NPR), as well as a GP cohort:

(i)TNFi cohort CRR (all countries): patients with PsA aged 18+ identified from CRR and initiating their first ever TNFi (adalimumab, certolizumab pegol, etanercept, golimumab, and infliximab) during the study period (index date).
(ii)non-TNFi bDMARD cohort CRR (all countries): patients with PsA aged 18+ identified from CRR and initiating their first ever non-TNFi bDMARD (abatacept, guselkumab, ixekizumab, kanakinumab, risankizumab, sekukinumab, and ustekinumab) during the study period (index date).
(iii)b/tsDMARD-naïve cohort CRR (all countries): patients with PsA aged 18+ identified from CRR naïve to b/tsDMARDs. The patients were indexed from the date of their first registered visit in the CRR during the study period.
(iv)Incident PsA cohort NPR (Denmark and Sweden): all individuals aged 18+ registered with ≥2 incident ICD-10 codes for PsA (M07.0-3 or L40.5) in the NPR of Denmark and Sweden during the study period, with at least 1 visit being from a department of rheumatology or internal medicine [45]. Patients were indexed from the date of their second registered PsA diagnosis. Patients with a recorded PsA visit before 2010 were excluded. This cohort is partly overlapping with the 3 CRR cohorts described above and also includes patients with PsA not registered in CRR for various reasons, eg, those with mild PsA disease. Also, the cohort only represents *incident* patients diagnosed with PsA between 2010 and 2021 and therefore is reflective of more contemporary PsA disease management and prognosis.
(v)GP cohort (Denmark and Sweden): for each incident patient with PsA from NPR (cohort 4 above) up to 5 individuals were randomly sampled from the GP matched on sex, year of birth, calendar year of start of follow-up, and residential area (for Sweden only). Individuals from this GP cohort were indexed on the same date as their index patient with PsA.

For all 5 cohorts, individuals with any prior invasive cancer or NMSC at the time of the cohort index date were excluded.

### Outcomes

The primary outcome was any type of incident NMSC (NMSC overall) and subtypes BCC and SCC identified from the National Cancer Registers according to ICD-10 and SNOMED codes. These 3 outcomes were investigated separately. For coding details, see Supplementary Table S1. Notably, for Finland and Norway, information on the NMSC subtype was not available.

### Follow-up

An ‘ever-treated’ definition constituted our main follow-up approach, corresponding to an intention-to-treat approach. Thus, all patients were followed from their index date until any of the dates of NMSC overall, BCC and SCC separately, an invasive non-NMSC cancer, death, emigration or end of study period, whichever came first. For the bDMARD-naïve cohort, follow-up also ended at any initiation of a b/tsDMARD. Consequently, for this main approach, each unique patient could contribute observations to several groups, and for the CRR cohorts 1 to 3, person-years (PY) of follow-up and outcomes could be attributed to both TNFi- and non-TNFi-treated cohorts.

### Covariates

For all 5 cohorts, we retrieved information on the following covariates (status at index date): age, sex, calendar year, country of birth (Nordic vs non-Nordic, as a proxy for skin type), highest attained educational level (as a proxy for socio-economic status), comorbid conditions such as cardiovascular disease, chronic obstructive pulmonary disease, diabetes mellitus, hypertension, and inflammatory bowel disease, comedication with csDMARDs, and oral glucocorticoids. We also collected the number of past hospitalisations at a rheumatology/internal medicine department, number of past hospitalisations at dermatological departments, as well as the presence of dermatological conditions (diagnosed cutaneous PsO and actinic keratosis). For the CRR cohorts 1 to 3, we additionally retrieved information on PsA disease duration, 28 Swollen Joint Count, 28 Tender Joint Count, general health patient on a 0 to 100 Visual Analogue Scale, Health Assessment Questionnaire (HAQ), C-reactive protein (CRP), disease activity score based on 28 joints and CRP (DAS28-CRP) at index date, as well as the number of previous b/tsDMARDs. We also collected information on lifestyle factors, ie, smoking habits and body mass index (BMI).

### Statistical analyses

We calculated descriptive statistics for the countries pooled and by country. For each cohort and country, crude incidence rates (IRs) per 100,000 PY and corresponding 95% CIs were calculated for each outcome.

In the CRR cohorts 1 to 3, we compared the incidence of NMSC, BCC, and SCC for the TNFi and the non-TNFi bDMARD cohorts with the b/tsDMARD-naïve cohort using a Cox regression model with attained age as the time scale, calculating hazard ratios (HRs). We adjusted for sex and calendar period (Model A) as well as for PsA disease duration on a continuous scale (Model B). In the incident PsA cohort from NPR (cohort 4), we compared the incidence and HR of NMSC, BCC, and SCC with the GP cohort (cohort 5) as the reference using a Cox regression model with attained age as the time scale and applying adjustments according to model A. All calculations were based on complete-case analyses, ie, no data were imputed, and Cox models were performed using only individuals with complete information on adjustment covariates.

To pool HRs across all countries, we used a random-effects meta-analysis approach. For CRR cohorts 1 to 3, we also calculated pooled exposure-adjusted numbers needed to harm (E-NNH) as the reciprocal of the difference in age- and sex-standardised IRs (std.IR) between TNFi/non-TNFi bDMARD cohorts and the b/tsDMARD-naïve cohort as reference. The E-NNH represented the PY of TNFi/non-TNFi bDMARD exposure needed for 1 excess event compared with b/tsDMARD-naïve, whereas an exposure-adjusted number needed to treat (E-NNT) was presented upon lower std.IRs in bDMARD-treated groups.

In all tables and figures, numbers of observed events below 3 (<3) were not displayed and grouped where applicable.

### Sensitivity analyses (CRR cohorts only)

To assess the impact of other bDMARD exposure time definitions on the risk of NMSC, we applied 2 alternative follow-up approaches among the CRR cohorts (analyses i and ii). To investigate the potential impact of MTX on NMSC risk, we also performed 2 additional sensitivity analyses (iii and iv) for the CRR cohorts based on data from Sweden and Denmark and using the main ‘ever-treated’ follow-up approach. Lastly, we performed a sensitivity analysis (v) with a calendar period cut-off from January 1, 2016 to December 31, 2021 to reflect a more contemporary bDMARD availability. The analysis was run with the main ‘ever-treated’ follow-up approach and for Denmark and Sweden only:

(i)A ‘most recent drug’ approach: Patients were followed from their index date until any date of initiation of a different type of b/tsDMARD (cohort censoring), outcome under investigation, any invasive non-NMSC cancer, death, emigration or end of study period, whichever came first. Patients who were switching from an originator to a biosimilar (or vice versa) were counted as remaining on the same treatment.
(ii)A ‘time-lagged most recent drug’ approach: Patients were followed from their index date + 90 days and until the date of initiating a different type of b/tsDMARD + 90 days (cohort censoring), outcome under investigation, any invasive non-NMSC cancer, death, emigration, or end of study period, whichever came first. For both of these sensitivity approaches, each unique patient could contribute observations to several groups, but PY of follow-up and outcomes could only be allocated to 1 treatment group at a time during follow-up.
(iii)A MTX-exposed criteria for b/tsDMARD-naïve PsA cohort was enforced, meaning that only those who had concomitant use of MTX at the index date were included in the b/tsDMARD-naïve PsA cohort.
(iv)In addition to the above, we stratified the TNFi cohort into patients who used TNFi as monotherapy and into those who used TNFi + concomitant MTX at the index date. TNFi monotherapy and TNFi + MTX, respectively, were compared with the b/tsDMARD-naïve MTX users.
(v)Same as the main analysis, but with a study period of January 1, 2016 to December 31, 2021.

## RESULTS

For Sweden and Denmark, we identified 23,553 incident patients with PsA from the NPRs contributing 129,522 PY (mean 5.5 years), with a corresponding 118,854 matched GP comparators contributing 657,375 PY (mean 5.3 years). Across all 4 Nordic countries, a total of 9999 TNFi-treated, 2200 non-TNFi bDMARD-treated, and 19,089 b/tsDMARD-naive patients with PsA were identified from CRRs. These 3 CRR cohorts contributed a total of 52,191 PY (mean 5.2 years), 7796 PY (mean 3.5 years), and 95,347 PY (mean 5.0 years), respectively. Baseline characteristics for all cohorts are presented in Table 1 (countries pooled together) and in Supplementary Tables S2 and S3 (by country). The age and sex distributions were comparable across all cohorts, although the TNFi-treated cohort was slightly younger (48.6 years), and the non-TNFi bDMARD cohort had a higher fraction of women (60.5%), Table 1. The non-TNFi bDMARD cohort had a higher DAS28-CRP, longer PsA disease duration, a higher number of previous b/tsDMARDs, and more comorbidities including PsO, at the start of follow-up, compared with both the TNFi and the b/tsDMARD-naïve cohorts.

**Table 1:** Baseline characteristics for patients with PsA from CRRs by treatment cohort (all countries pooled together) as well as for incident patients with PsA from the NPR and their age-, sex-, and calendar year-matched GP comparator (Denmark and Sweden pooled together)

	Patients with PsA from CRR by treatment cohort (all countries)			Patients with PsA from NPR and matched GP cohort (Sweden and Denmark)	
Variable	TNF inhibitor, N	Non-TNFi bDMARD, N	b/tsDMARD-naïve, N	Incident PsA, N	GP, N
No. of individuals in each cohort	9999	2200	19,089	23,553	118,854
Treatment distribution by country					
Denmark	1918	415	8042	5125	25 873
Finland	591	130	1540	-	-
Norway	950	89	192	-	-
Sweden	6540	1566	9315	18,428	92,981
Women, (%)	5228 (52.3)	1332 (60.5)	10,013 (52.5)	12,926 (54.9)	65,115 (54.8)
Age in y, (%)	48.6 (13.1)	51.0 (12.4)	51.3 (14)	51.3 (14.9)	51.1 (14.7)
Country of birth, (%)					
Nordic	7863 (78.6)	1838 (83.5)	16,363 (85.7)	21,882 (92.9)	99,538 (83.7)
Non-Nordic	595 (6.0)	143 (6.5)	994 (5.2)	1671 (7.1)	19,316 (16.3)
Educational level, (%)					
≤9 y	1457 (14.6)	348 (15.8)	3816 (20.0)	4340 (18.6)	21,391 (18.3)
10-12 y	3423 (34.2)	745 (33.9)	7501 (39.3)	8228 (35.2)	36,411 (31.1)
>12 y	4552 (45.5)	1010 (45.9)	7053 (37.0)	10,774 (46.2)	59,142 (50.6)
PsA disease duration higher than 5 y, (%)	5442 (54.4)	1577 (71.7)	6525 (34.2)	-	-
No. of previous b/tsDMARDs, (%)					
0	9447 (94.5)	341 (15.5)	19,089 (100)	-	-
1	424 (4.2)	614 (27.9)	0 (0)	-	-
2+	118 (1.2)	1242 (56.5)	0 (0)	-	-
Calendar year (range)					
Denmark	2010-2021	2010-2021	2010-2021	2010-2021	2010-2021
Finland	2010-2018	2010-2018	2010-2018		
Norway	2010-2019	2014-2019	2010-2012		
Sweden	2010-2021	2010-2021	2010-2021	2010-2021	2010-2021
PsA-related characteristics, mean (SD)					
DAS28-CRP	3.8 (1.1)	3.9 (1.2)	3.3 (1.2)	-	-
Missing DAS28-CRP	4195 (42)	1063 (48.3)	7992 (41.9)	-	-
CRP (mg/L)	10 (17)	10.8 (17.7)	9.4 (15.6)	-	-
VAS general health patient (0-100)	57.1 (24.0)	63 (24.2)	47.2 (27.5)	-	-
HAQ score (0-3)	0.8 (0.6)	1 (0.6)	0.7 (0.5)	-	-
Swollen joint count (0-28)	2.7 (3.4)	2.5 (3.6)	2 (3.1)	-	-
Tender joint count (0-28)	5 (5.4)	5.6 (6.1)	3.6 (4.7)	-	-
Concomitant treatments, (%)					
Any csDMARD	4614 (46.2)	656 (29.8)	12,504 (65.5)	9539 (40.5)	538 (0.5)
MTX	3977 (39.8)	554 (25.2)	10,903 (57.1)	7982 (33.9)	234 (0.2)
Prednisolone	1258 (12.6)	308 (14)	2229 (11.7)	4185 (17.8)	1163 (1)
Comorbidities, (%)					
Cardiovascular disease	412 (4.1)	122 (5.5)	934 (4.9)	1120 (4.8)	3336 (2.8)
COPD	123 (1.2)	53 (2.4)	316 (1.7)	637 (2.7)	1635 (1.4)
Diabetes mellitus	557 (5.6)	176 (8.0)	993 (5.2)	1730 (7.3)	4746 (4)
Hypertension	957 (11.3)	334 (16.9)	1907 (11.0)	2955 (12.5)	7557 (6.4)
Inflammatory bowel disease	153 (1.8)	37 (1.9)	210 (1.2)	335 (1.4)	788 (0.7)
No. of hospitalisations at rheumatology/internal medicine department, mean (SD)	0.4 (1.2)	0.5 (1.4)	0.9 (1.3)	0.5 (1.1)	0.1 (0.6)
Dermatological comorbidity, (%)					
Cutaneous psoriasis	4989 (49.9)	1379 (62.7)	7645 (40.0)	11,847 (50.3)	1750 (1.5)
Actinic keratosis	91 (0.9)	27 (1.2)	167 (0.9)	284 (1.2)	1107 (0.9)
No. of hospitalisations at dermatologic department, mean (SD)	0.1 (0.5)	0.1 (0.6)	0.1 (0.8)	0.1 (0.6)	0 (0.2)
Lifestyle factors					
Smoking, (%)					
Current	1038 (10.4)	318 (14.5)	1798 (9.4)	-	-
Former	1874 (18.7)	607 (27.6)	2756 (14.4)	-	-
Never	2709 (27.1)	719 (32.7)	4614 (24.2)	-	-
Missing	4378 (43.8)	556 (25.3)	9921 (52.0)	-	-
BMI (kg/m^2^), mean (SD)	28.5 (6.1)	28.8 (6.0)	28.3 (6.6)	-	-
Missing	9058 (90.6)	2011 (91.4)	16,761 (87.8)	-	-

bDMARD, biological disease-modifying antirheumatic drug; BMI, body mass index; b/tsDMARD-naïve, No exposure to a bDMARD or a targeted synthetic DMARD; COPD, chronic obstructive pulmonary disease; CRP, C-reactive protein; CRR, Clinical Rheumatology Register; csDMARD, conventional synthetic disease-modifying antirheumatic drug; DAS28-CRP, Disease Activity Score in 28 joints; GP, general population; HAQ, Health Assessment Questionnaire; MTX, methotrexate; N, Number of; non-TNFi bDMARD, encompasses abatacept, guselkumab, ixekizumab, kanakinumab, risankizumab, sekukinumab, and ustekinumab; NPR, National Patient Registry; PsA, psoriatic arthritis; TNFi, tumour necrosis factor inhibitor; VAS, visual analogue scale.

### Risk of NMSC in incident patients with PsA vs the GP

**Figure 1:**
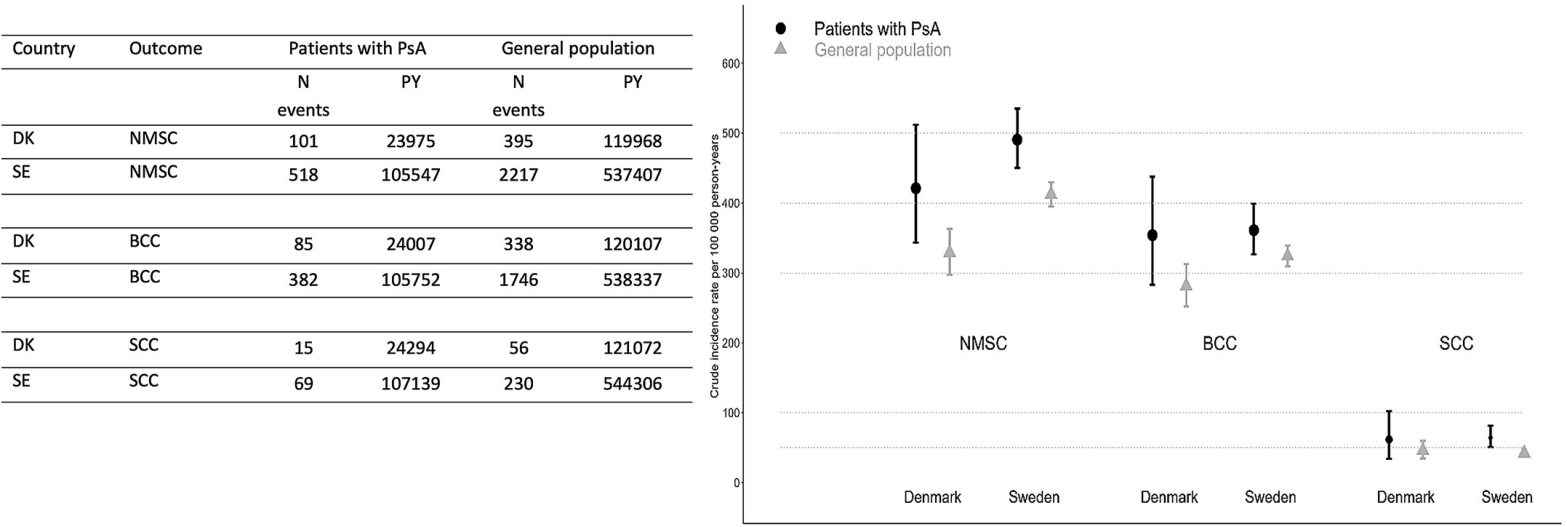
Crude incidence rates (per 100,000 PY) of NMSC, BCC, and SCC in incident patients with PsA from NPR vs the general population and presented by country. The number of events, person-years (PY), and abbreviations are displayed in the adjacent table. BCC, basal cell carcinoma; NMSC, nonmelanoma skin cancer; NPR, National Patient Register; PsA, psoriatic arthritis; SCC, squamous cell carcinoma.

**Figure 2:**
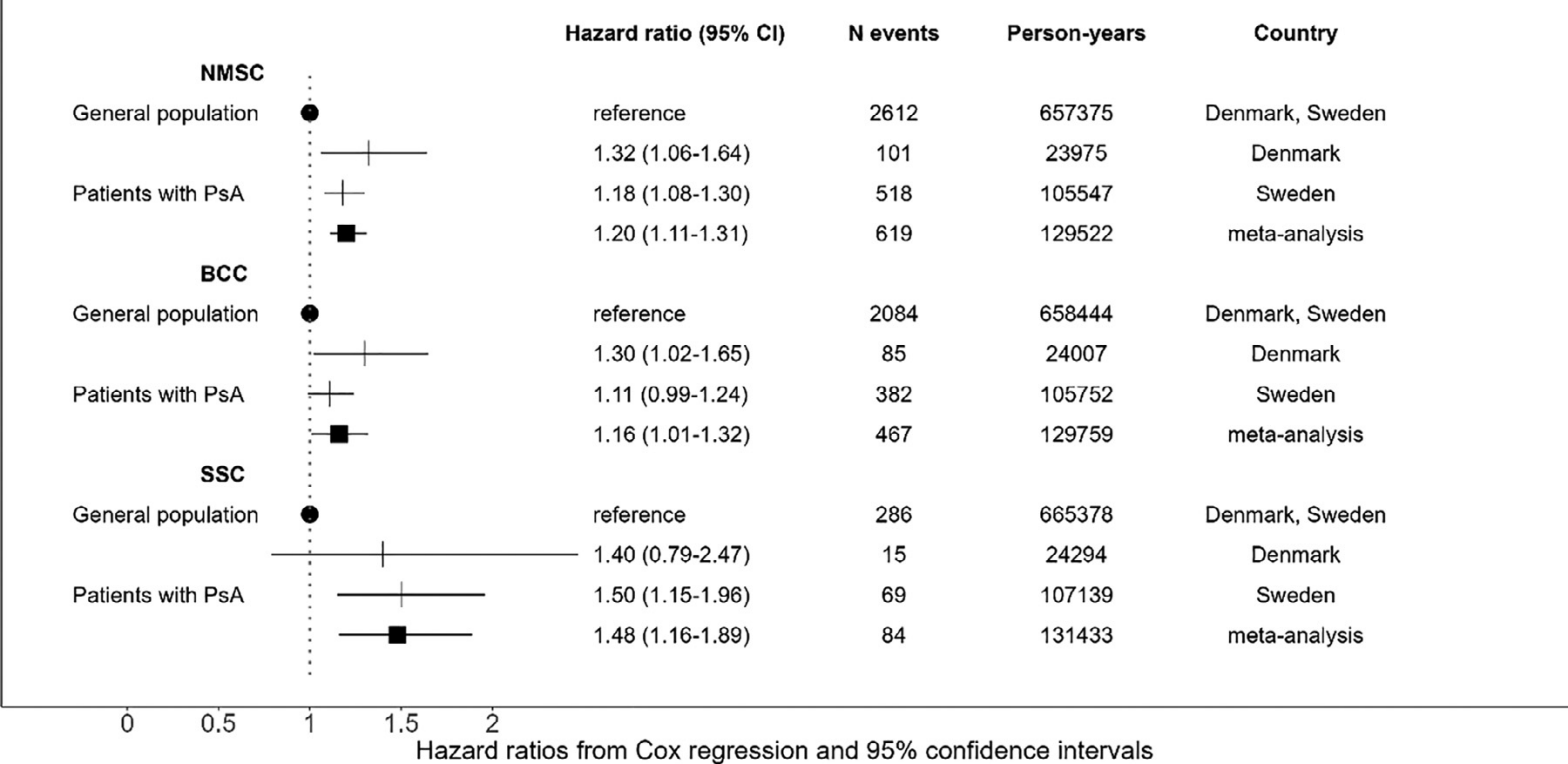
Incident patients with PsA from NPR vs the general population by country and DK+SE pooled together by meta-analysis, adjusted Cox model A. BCC, basal cell carcinoma; bDMARD, biological disease-modifying antirheumatic drug; b/tsDMARD-naïve, no exposure to a bDMARD or a targeted synthetic DMARD; DK, Denmark; N, Number of; NMSC, nonmelanoma skin cancer; NPR, National Patient Register; SCC, squamous cell carcinoma; SE, Sweden; TNFi, tumour necrosis factor inhibitor; non-TNFi bDMARD, encompasses abatacept, guselkumab, ixekizumab, kanakinumab, risankizumab, sekukinumab, and ustekinumab; PsA, psoriatic arthritis. General population: age-, sex-, and calendar year-matched to the incident PsA patients. Cox model A: adjustment for sex and calendar period, and with attained age as the underlying time scale.

From Sweden and Denmark, we identified 619 NMSCs among patients with incident PsA and 2612 NMSCs in the GP comparator, Figure 1. The pooled crude IRs for NMSCs overall were 478 (incident PsA cohort) and 397 (GP cohort) per 100,000 PY, with country-specific crude IRs of 421 (Denmark) and 491 (Sweden) among incident PsA. This resulted in a pooled HR (Cox model A) for NMSC of 1.20 (95% CI: 1.11-1.31) in PsA compared with the GP, Figure 2. The corresponding HRs were 1.16 (95% CI: 1.01-1.32) for BCC and 1.48 (95% CI: 1.16-1.89) for SCC.

### Risk of NMSC in bDMARD-treated versus b/tsDMARD-naïve PsA patients

**Figure 3:**
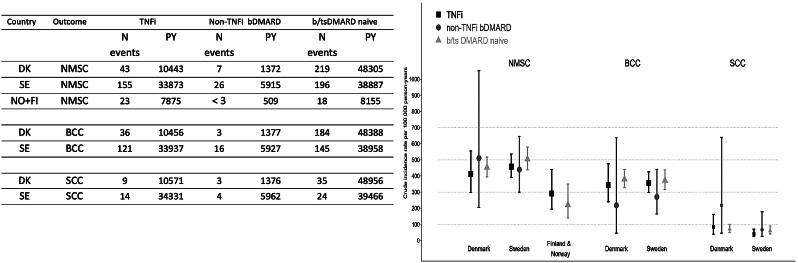
Crude incidence rates (per 100,000 PY) of NMSC, BCC, and SCC in patients with PsA identified in CRRs and presented by country (except for pooled NO and FI). The number of events, person-years (PY), and abbreviations are displayed in the adjacent table. BCC, basal cell carcinoma; bDMARD, biological disease-modifying antirheumatic drug; b/tsDMARD-naïve, No prior exposure to a bDMARD or a targeted synthetic DMARD; CRR, Clinical Rheumatology Register; NMSC, nonmelanoma skin cancer; PsA, psoriatic arthritis; SCC, squamous cell carcinoma; TNFi, tumour necrosis factor inhibitor.

**Figure 4:**
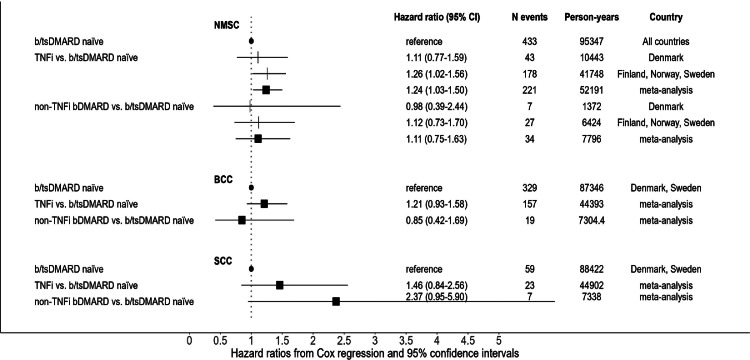
Pooled results of NMSC overall, BCC, and SCC in patients with PsA identified in CRRs for DK and NO+FI+SE, adjusted Cox model. BCC, basal cell carcinoma; bDMARD, biological disease-modifying antirheumatic drug; b/tsDMARD-naïve, No prior exposure to a bDMARD or a targeted synthetic DMARD; CRR, Clinical Rheumatology Register; DK, Denmark; FI, Finland; N, Number of; NMSC, nonmelanoma skin cancer; NO, Norway; non-TNFi bDMARD, encompasses abatacept, guselkumab, ixekizumab, kanakinumab, risankizumab, sekukinumab, and ustekinumab; PsA, patients; SCC, squamous cell carcinoma; SE, Sweden; TNFi, tumour necrosis factor inhibitor. Cox model B: adjustment for sex, calendar period, and PsA disease duration, and with attained age as the underlying time scale.

We identified a total of 688 events of NMSC overall among patients with PsA from CRRs in all countries: 221 for TNFi, 34 for non-TNFi bDMARD, and 433 for the b/tsDMARD-naïve cohort, Figure 3. This corresponded to pooled crude IRs per 100,000 PY of 423 (TNFi cohort), 436 (non-TNFi bDMARD cohort), and 454 (b/tsDMARD-naïve). As few events were observed for both Norway and Finland, these countries were combined for all country-specific outcome analyses. Using Cox model B, pooled HRs for all countries were 1.24 (95% CI: 1.03-1.50) with TNFi and 1.11 (95% CI: 0.75-1.63) with non-TNFi bDMARDs compared with b/tsDMARD-naïve PsA, Figure 4. The age- and sex-std.IRs demonstrated a higher incidence of NMSC among TNFi-treated (std.IR = 557/100,000 PY) and non-TNFi-treated (std.IR = 575/100,000 PY) compared with b/tsDMARD-naïve PsA (std.IR = 459/100,000 PY). This corresponded to an E-NNH of 1020 and 862, respectively, Table 2. Sensitivity analyses using alternative follow-up approaches did not markedly alter the HRs for NMSC overall, although the 95%CIs for TNFi vs b/tsDMARD-naive now crossed unity, Supplementary Tables S4 and S5, and neither did the sensitivity analysis using a restricted calendar period from January 1, 2016 to December 31, 2021, Supplementary Tables S6 and S7.

**Table 2:** Pooled exposure-adjusted number needed to harm of NMSC overall, BCC, and SCC in patients with PsA identified in all 4 Nordic CRRs, based on age- and sex-standardised IRs

Cohort	Outcome	PY	N events	Crude IR (per 100,000 PY)	Age- and sex-standardised IR (per 100,000 PY)	E-NNH (PY) based on age- and sex-standardised IRs
b/tsDMARD-naive (Ref.)	NMSC	95,347	433	454	459	Ref.
TNFi	NMSC	52,191	221	423	557	1020
Non-TNFi bDMARD	NMSC	7796	34	436	575	862
b/tsDMARD-naive (Ref.)	BCC	87,346	329	377	376	Ref.
TNFi	BCC	44,393	157	354	468	1087
Non-TNFi bDMARD	BCC	7304	19	270	304	1389^a^
b/tsDMARD-naive (Ref.)	SCC	88,422	59	67	66	Ref.
TNFi	SCC	44,902	23	51	79	7692
Non-TNFi bDMARD	SCC	7338	7	95	210	694

BCC, basal cell carcinoma; bDMARD, biological disease-modifying antirheumatic drug; b/tsDMARD-naïve, No exposure to a bDMARD or a targeted synthetic DMARD; CRR, Clinical Rheumatology Register; E-NNH, exposure-adjusted numbers needed to harm; IR, incidence rate; N, Number of; NMSC, nonmelanoma skin cancer; NNH, numbers needed to harm; non-TNFi bDMARD, encompasses abatacept, guselkumab, ixekizumab, kanakinumab, risankizumab, sekukinumab, and ustekinumab; PsA, psoriatic arthritis; PY, person-years; Ref., reference; SCC, squamous cell carcinoma; TNFi, tumour necrosis factor inhibitor.

Exposure-adjusted number needed to harm (E-NNH) PY was calculated as the reciprocal of the difference in age- and sex-standardised IRs between TNFi/non-TNFi bDMARD cohorts and the b/tsDMARD-naïve cohort as the reference.

a Exposure-adjusted number needed to treat (E-NNT) instead of NNH.

For NMSC subtypes BCC/SCC (only available for Sweden and Denmark), we identified a total of 157/23 events for TNFi, 19/7 for non-TNFi bDMARD, and 329/59 BCC and SCC events for b/tsDMARD-naïve cohorts, respectively, Figure 3. As expected, the pooled crude IRs were much higher for BCC (ranging between 260 and 377) than for SCC (ranging between 51 and 95) across all 3 CRR cohorts. For BCC, the pooled analyses showed an adjusted HR (Cox model B) of 1.21 (95% CI: 0.93-1.58) for TNFi and 0.85 (95% CI: 0.42-1.69) for non-TNFi bDMARDs compared with b/tsDMARD-naïve. For SCC, the corresponding adjusted HRs were 1.46 (95% CI: 0.84-2.56) for TNFi and 2.37 (95% CI: 0.95-5.90) for non-TNFi bDMARDs, Figure 4. E-NNH of 1087 (TNFi) and E-NNT of 1389 (non-TNFi bDMARD) for BCC were found using b/tsDMARD-naïve patients as the reference, and the E-NNH for SCC were 7692 (TNFi) and 694 (non-TNFi bDMARD), Table 2. Sensitivity analyses using alternative follow-up approaches showed HRs similar to the ever-treated approach of the main analysis, but with the HR for SCC slightly amplified in the ‘time-lagged most recent drug’ approach: 3.43 (95% CI: 1.25-9.39), Supplementary Tables S4 and S5.

### MTX sensitivity analyses

Concomitant use of MTX at index among the b/tsDMARD-naïve PsA comparators did not substantially alter the HRs of NMSC for TNFi or for non-TNFi bDMARDs. In addition, stratifying the TNFi-treated PsA cohort into patients who received TNFI monotherapy or TNFI + concomitant MTX did not show higher HRs among TNFI + MTX users, Supplementary Table S8.

## DISCUSSION

In this large collaborative registry-based observational study of patients with PsA from 4 Nordic countries, we found increased risks of NMSC overall, BCC, and SCC in incident PsA compared with the GP. We also found that TNFi-treated patients with PsA experienced an increased risk of NMSC overall compared with b/tsDMARD-naïve patients. For non-TNFi bDMARD-treated patients with PsA, there were no statistically significant differences in risk of NMSC overall, nor for BCC/SCC individually, but a more than doubled HR of SCC was seen compared with b/tsDMARD-naïve patients.

In our pooled analyses of contemporary incident patients with PsA from Sweden and Denmark, we found an increased risk for NMSC overall compared with an age-, sex-, and calendar year-matched GP: HR 1.20, 95% CI: 1.11 to 1.31. The corresponding HRs for BCC and SCC were 1.16 (95% CI: 1.01-1.32) and 1.48 (95% CI: 1.16-1.89), respectively. These findings suggest that PsA itself carries an intrinsic risk of both BCC and even more for SCC. An underlying increased risk of NMSC in patients with PsA/PsO has been described in other studies [3,4,10]. The systematic review and meta-analysis by Vaengebjerg et al [4] from 2020 found a risk ratio of 1.22 (95% CI: 0.89-1.66) in PsA vs GP, whereas a Danish cohort study from 2016 found an IR ratio of 1.62 (95% CI: 1.27-2.05) [3]. Furthermore, our study results align with those of Wang et al [10], showing that patients with PsO (mixed PsO and PsA population) had a higher relative risk (RR) for SCC (RR: 2.08, 95% CI: 1.53-2.83) than for BCC (RR: 1.28, 95% CI: 0.81-2.00) compared with those without PsO. However, despite our analyses consisting of only incident patients with PsA, the potential impact of pre-existing PsO disease at baseline (50.3% incident PsA cohort vs 1.5% GP cohort) has to be considered before attributing excess NMSC risk to PsA disease itself.

Some previous studies have indicated an increased NMSC risk with TNFi in patients with PsO/PsA [5,18,22,23,[26], [27], [28]]. For PsA specifically, the systematic review and meta-analysis by Wu et al [5] from 2025 found a standardised incidence ratio of 1.84 (95% CI: 1.160-2.916) for any type of NMSC in TNFi-treated PsA compared with the GP. The present study’s pooled analyses for NMSC overall also showed an increased risk with TNFi: HR 1.24 (95% CI: 1.03-1.50) compared with b/tsDMARD-naïve PsA. That said, direct comparisons of the 2 studies’ risk estimations are challenged by differing comparators; the study by Wu et al [5] carries within a potential PsA-intrinsic NMSC risk when comparing with the GP, which complicates TNFi-specific inferences. To our knowledge, our present study is 1 of the first to investigate TNFi-related risk of NMSC within a population consisting solely of patients with PsA. Also, our pooled analyses showed HRs of 1.21 (95% CI: 0.93-1.58) and 1.46 (95% CI: 0.84-2.56) for BCCs and SCCs, respectively, for TNFi compared with b/tsDMARD-naïve PsA. These results suggest that—although not statistically significant—the TNFi-related NMSC risk pertains to both BCC and SCC, but may be most pronounced for SCC. In absolute terms, it is also important to remember that the E-NNH for NMSC (=1020) meant that 1020 PsA patients required 1 PY of TNFi treatment before observing 1 excess NMSC. Interestingly, use of concomitant MTX did not substantially alter the results of our main analysis. If anything, HRs for NMSC overall, BCC, and SCC were higher for TNFi monotherapy than for TNFi + MTX, indicating that MTX itself does not potentiate the TNFi-related NMSC risk in patients with PsA. From a clinical perspective, this finding carries important implications.

For non-TNFi bDMARD, we found no increased risks for NMSC overall (HR: 1.11, 95% CI: 0.75-1.63) or for BCC (HR: 0.85, 95% CI: 0.42-1.69) when compared with b/tsDMARD-naïve PsA. Looking at SCC specifically, the corresponding HR was 2.37 (95% CI: 0.95-5.90) for non-TNFi bDMARDs—a signal that persisted in sensitivity analyses. For contextualisation on an absolute scale, the E-NNH of 694 for SCC was still quite high, though when comparing non-TNFi bDMARDs with b/tsDMARD-naïve PsA. A particular susceptibility to SCC development for PsO/PsA patients compared with the GP is in line with other studies as well as the present study [5,10], but whether such SCC risk is potentially amplified with non-TNFi bDMARD use in PsA has to our knowledge only been investigated in few other studies [18,24]. Of these, most primarily included patients with PsO and found no increased risk with non-TNFi bDMARDs; the observational study of patients with PsO by deShazo et al [18] even observed a decreased HR of 0.30 (95% CI: 0.10-0.90) with ustekinumab compared with bDMARD/MTX-naïve patients. Of note, our non-TNFi bDMARD cohort consisted of drugs with different modes of action (MoA), which makes it difficult to draw MoA-specific inferences in terms of NMSC risk; such MoA-specific risks of NMSC, including subtypes BCC and SCC, need to be investigated in future studies when power allows for it.

There are study limitations to be mentioned. Patients with PsA and active PsO probably see a dermatologist—who is trained at diagnosing NMSC—at least once a year, which makes surveillance bias a potential problem in GP comparisons. A similar argument could be made for bDMARD-treated PsA in case of a higher number of dermatologist visits compared with the b/tsDMARD-naïve. Further, despite accounting for differences in age, sex, calendar period, and PsA disease duration for the CRR treatment cohorts, we cannot rule out the presence of residual confounding by indication, ie, if the extent and severity of PsA or underlying PsO disease activity itself was related to the magnitude of NMSC risk while also influencing the choice of treatment. However, modelling adjustment for baseline-captured measurements reflective of PsA disease activity such as DAS28-CRP was refrained from in our study setting with long follow-up time. Instead, we adjusted for PsA disease duration, which is better reflective of previous cumulative disease activity. Unfortunately, information on PsO Area and Severity Index scores and phototherapy was not available, and a limitation regarding the lack of information on NMSC subtypes from Norway and Finland must also be mentioned. Furthermore, our study included high proportions of missingness on lifestyle factors such as smoking and BMI, as well as on PsA-related characteristics such as DAS28-CRP and HAQ. Lastly, although death was a competing risk not accounted for in our study and a potential limitation, we considered that the expected magnitude (cumulative incidence) of death was unlikely to greatly impact the study results and interpretations thereof in our relatively ‘young’ study population of patients with PsA.

Strengths of the study included the ability to identify and pool patients with PsA from 4 Nordic countries. Using stringent PsA exposure definitions in Danish and Swedish NPRs, we were able to assess the underlying risk of NMSC overall and BCC/SCC individually in a population consisting of all contemporary incident patients with PsA. Also, nationwide identification of PsA diagnoses and bDMARD treatments in Nordic CRRs as well as NMSCs via NCRs provided our study with highly valid and complete exposure and outcome definitions at an individual-based level. In contrast to other studies of NMSC risk in bDMARD-treated PsA/PsO individuals compared with those without PsA/PsO, we were also able to assess risk of NMSC with TNFi and non-TNFi bDMARDs solely within a PsA population, thereby attempting to refine treatment-specific effects from those of PsA itself.

In conclusion, we found increased risks of NMSC overall, BCC, and SCC in patients with PsA compared with the GP—a signal most pronounced for SCC. Treatment with TNFi was associated with a 24% increased hazard for NMSC overall relative to b/tsDMARD-naïve patients, which on the absolute scale corresponded to an E-NNH of 1020. Conversely, non-TNFi bDMARDs were not associated with increased risks of NMSC overall nor BCC, but a numerically more than doubled hazard of SCC was found vs b/tsDMARD-naïve (E-NNH of 694). Caveats remain regarding the causality of the relationship between bDMARD use and the development of NMSCs in PsA, as we cannot rule out the presence of surveillance bias and residual confounding by indication. Irrespective of causality, contextualisation of excess NMSC risk on the absolute scale is warranted when considering NMSC surveillance strategies of bDMARD-treated patients with PsA in clinical practice.

## CRediT authorship contribution statement

**Rasmus Westermann:** Writing – review & editing, Writing – original draft, Visualization, Validation, Supervision, Software, Resources, Project administration, Methodology, Investigation, Funding acquisition, Formal analysis, Data curation, Conceptualization. **Bénédicte Delcoigne:** Writing – review & editing, Writing – original draft, Visualization, Validation, Supervision, Software, Resources, Project administration, Methodology, Investigation, Funding acquisition, Formal analysis, Data curation, Conceptualization. **René Lindholm Cordtz:** Writing – review & editing, Writing – original draft, Visualization, Validation, Supervision, Software, Resources, Methodology, Investigation, Formal analysis, Data curation, Conceptualization. **Sella Aarrestad Provan:** Writing – review & editing, Validation, Resources, Project administration, Methodology, Investigation, Formal analysis, Data curation. **Joe Sexton:** Writing – review & editing, Validation, Software, Resources, Methodology, Investigation, Formal analysis, Data curation, Conceptualization. **Dan Nordström:** Writing – review & editing, Validation, Resources, Methodology, Investigation, Funding acquisition, Formal analysis, Data curation, Conceptualization. **Pia Isomäki:** Writing – review & editing, Validation, Software, Resources, Methodology, Investigation, Formal analysis, Data curation, Conceptualization. **Johan Askling:** Writing – review & editing, Validation, Supervision, Resources, Project administration, Methodology, Investigation, Funding acquisition, Formal analysis, Data curation, Conceptualization. **Lene Wohlfahrt Dreyer:** Writing – review & editing, Validation, Supervision, Resources, Project administration, Methodology, Investigation, Funding acquisition, Formal analysis, Data curation, Conceptualization. **Karin Hellgren:** Writing – review & editing, Writing – original draft, Visualization, Validation, Supervision, Software, Resources, Project administration, Methodology, Investigation, Funding acquisition, Formal analysis, Data curation, Conceptualization.

## Appendix Supplementary materials

Image, application 1

## Footnotes

Supplementary material associated with this article can be found in the online version at doi:10.1016/j.ero.2026.03.009.
