# Supplementary material for: Risk of nonmelanoma skin cancer in patients with psoriatic arthritis and according to bDMARD treatment—a cohort study from the Nordic Arthritis Research Collaboration
Source: EULAR Rheumatol Open. 2026 Apr 9;2(2):100161. doi: 10.1016/j.ero.2026.03.009 (PMC13425213; doi:10.1016/j.ero.2026.03.009)
Supplement: Supplementary file 1 [file mmc1.docx]

**Supplementary file:** *Risk of non-melanoma skin cancer in patients with psoriatic arthritis and according to bDMARD treatment – A cohort study from the Nordic Arthritis Research Collaboration*

**Supplementary Table S1.** Variable definitions and data sources

| Variable | Reporting of variable | | | ICD10, SNOMED, ATC or surgical procedure codes | Registration period, data source and definition | |
| --- | --- | --- | --- | --- | --- | --- |
| Definition of PsA diagnosis | - | | | L405, M070, M071, M072, M073 | CRR cohorts: as registered in the CRRs in any of the countries  *or*  Incident PsA cohort NPRs: ≥2 recorded diagnoses in Danish or Swedish NPRs, with at least one visit from an internal medicine or rheumatology department | |
| b/tsDMARD treatments: | - | | | Start and stop dates | Collected from the CRRs contain information on b/tsDMARD treatments including start and stop dates | |
| Demographics and education disease related characteristics |  | | |  |  | |
| Male, n (%) | n (%) | | | - | Recorded at start of follow-up for all study participants in the CRRs  Recorded at start of follow-up for all study participants in population registers | |
| Age (years) | mean (IQR) | | | - |  |  |
| Education level | n (%) | | | - |  |  |
| Country of birth | Nordic, Non-Nordic, N (%) | | |  |  |  |
| PsA-disease related characteristics |  | | |  |  | |
| Tender joint count (0-28) | median (IQR) [missing] | | - | | | Recorded at start of follow-up (window: 8 weeks before until 3 weeks after) for all study participants in the CRRs for all countries. The value closest to the start of follow-up was chosen.      Recorded at start of from CRRs and/or in national prescription drug registers |
| Swollen joint count (0-28) | median (IQR) [missing] | | - | | |  |
| VAS general health patient  (0-100) | median (IQR) [missing] | | - | | |  |
| HAQ score (0-3) | median (IQR) [missing] | | - | | |  |
| DAS28-CRP (0-10) | median (IQR) [missing] | | - | | |  |
| CRP (mg/liter) | median (IQR) [missing] | | - | | |  |
| Concomitant use of  csDMARDs or/and oral  steroids | n (%) | | - | | |  |
| PsA disease duration (years) | median (IQR) [missing] | | - | | | Recorded in CRRs by the treating physician |
| Cutaneous psoriasis | yes/no, n (%) | | ICD10 L40  (except L 40.5)  and/ or  ATC code D05 | | | Recorded with a diagnosis of psoriasis in NPR any time before start of follow up. |
| Lifestyle factors | | | | | | |
| BMI (kg/m^2^) | median (IQR) [missing] | | - | | | Recorded in the clinical rheumatology registers within 1 year before start of follow-up. The value closest to the follow-up start date was chosen. |
| Smoking status, n (%) | Current, previous, never, missing, n (%) | | - | | |  |
| Comorbidities | | | | | | |
| Cardiovascular disease | n (%) | | I20-I25, I50, I60-I69 | | | Recorded up to 5 years prior to start of follow-up in the NPRs of each country |
| Chronic obstructive  pulmonary disease (COPD) | n (%) | | J41-J44 | | |  |
| Diabetes mellitus | n (%) | | E10-E14 | | |  |
| Hypertension | n (%) | | I10-I13, I15 | | |  |
| Inflammatory bowel disease | n (%) | | K50-K51 | | |  |
| No. of hospitalizations | median (range) | | Registered hospitalizations in an internal medicine department | | |  |
| No. of dermatological  hospitalizations | median (range) | | Registered hospitalizations in a dermatological department | | |  |
| Actinic keratosis | n (%) | | ICD-10: L57.0 | | |  |
| NMSC |  | |  | | |  |
| Non-melanoma skin cancer (NMSC)  overall | | - | ICD10 C44 (including basal cell carcinomas, squamous cell carcinomas, *and* other subtypes of NMSC) | | | Recorded in the national cancer registers of each country.  Note: for Sweden all events of BCC were identified in the Swedish basal cell cancer register. |
|  | |  |  | | |  |
| Basal cell carcinoma (BCC)  Squamous cell carcinoma (SCC) | |  | ICD10 C44 + SNOMED 8090/3 BCC, NOS 8091/3 BCC, multifocal, superficial 8092/3 BCC, infiltrating 8093/3 BCC, fibroepithelial 8097/3 BCC, nodular 8123/3 BCC, basaloid carcinoma  ICD10 C44 + SNOMED 8073/3 SCC, squamous cell carcinoma | | |  |
| Abbreviations PsA: Psoriatic Arthritis, b/tsDMARD: biological and targeted synthetic disease modifying anti-rheumatic drug, CRRs: Clinical Rheumatology Registers, NPRs: National Patient Registers, IQR: interquartile range, BMI: body mass index, CRP: C-reactive protein, VAS: visual analogue scale, HAQ: Health Assessment Questionnaire, DAS28-CRP: Disease Activity Score in 28 joints. | | | | | | |

**Supplementary Table S2.** Baseline characteristics for patients with PsA from all four Nordic CRRs by treatment cohort and by country

| Cohort | TNFi, N | | | | non-TNFi bDMARD, N | | | | b/tsDMARD-naïve, N | | | |
| --- | --- | --- | --- | --- | --- | --- | --- | --- | --- | --- | --- | --- |
| Country | dk | fi | no | se | dk | fi | no | se | dk | fi | no | se |
| N individuals in each cohort | 1918 | 591 | 950 | 6540 | 415 | 130 | 89 | 1566 | 8042 | 1540 | 192 | 9315 |
| Women, (%) | 1091 (56.9) | 277 (46.9) | 515 (54.3) | 3345 (51.1) | 262 (63.1) | 69 (53.1) | 62 (69.7) | 939 (60.0) | 4431 (55.1) | 786 (51.0) | 93 (48.4) | 4703 (50.5) |
| Age in years, (%) | 47.2 (13) | 48 (12) | 47 (14) | 49 (13) | 48.4 (13.2) | 50 (12) | 54 (11) | 52 (12) | 50.2 (13.8) | 50 (14) | 45 (18) | 52 (14) |
| Country of birth, (%) |  |  |  |  |  |  |  |  |  |  |  |  |
| Nordic | 1801 (93.9) | n/a | n/a | 1449 (92.5) | 389 (93.7) | n/a | n/a | 8705 (93.5) | 7658 (95.2) | n/a | n/a | 6062 (92.7) |
| Non-Nordic | 117 (6.1) | n/a | n/a | 117 (7.5) | 26 (6.3) | n/a | n/a | 610 (6.5) | 384 (4.8) | n/a | n/a | 478 (7.3) |
| Educational level, (%) |  |  |  |  |  |  |  |  |  |  |  |  |
| ≤9 years | 432 (22.5) | 34 (5.8) | 90 (9.5) | 901 (13.8) | 115 (27.7) | 12 (9.2) | 9 (10.1) | 212 (13.5) | 1919 (23.9) | 250 (16.2) | 24 (12.5) | 1623 (17.4) |
| 10-12 years | 938 (48.9) | 97 (16.4) | 340 (35.8) | 2048 (31.3) | 198 (47.7) | 23 (17.7) | 21 (23.6) | 503 (32.1) | 4098 (51.0) | 308 (20.0) | 124 (64.6) | 2971 (31.9) |
| >12years | 516 (26.9) | 122 (20.6) | 365 (38.4) | 3549 (54.3) | 96 (23.1) | 31 (23.8) | 38 (42.7) | 845 (54.0) | 1918 (23.8) | 411 (26.7) | 41 (21.4) | 4683 (50.3) |
| PsA disease duration higher than 5 years, (%) | 647 (33.7) | 259 (43.8) | 602 (88.9) | 3934 (60.2) | 221 (53.3) | 83 (63.8) | 58 (96.7) | 1215 (78.0) | 1430 (17.8) | 369 (24.0) | 192 (100.0) | 4534 (48.7) |
| N of previous b/tsDMARDs, (%) |  |  |  |  |  |  |  |  |  |  |  |  |
| 0 | 1882 (98.1) | 543 (91.9) | 668 (70.3) | 6354 (97.2) | 69 (16.6) | 29 (22.3) | < 5 | 239 (15.3) | 8042 (100.0) | 1540 (100.0) | 192 (100.0) | 9315 (100.0) |
| 1 | < 40 | 41 (6.9) | 167 (17.6) | 176 (2.7) | 112 (27.0) | 38 (29.2) | 16 (18.0) | 448 (28.6) | 0 (0.0) | 0 (0.0) | 0 (0.0) | 0 (0.0) |
| 2+ | < 3 | 7 (1.2) | 98 (10.3) | 10 (0.2) | 234 (56.4) | 63 (48.5) | 66 (74.2) | 879 (56.1) | 0 (0.0) | 0 (0.0) | 0 (0.0) | 0 (0.0) |
| **PsA-related characteristics, mean (SD)** |  |  |  |  |  |  |  |  |  |  |  |  |
| DAS28-CRP | 3.8 (1.2) | 3.5 (1.2) | 3.5 (1.1) | 4.0 (1.1) | 3.9 (1.4) | 3.6 (1.1) | 3.7 (1.2) | 4.0 (1.2) | 3.4 (1.3) | 3.0 (1.1) | 3.7 (1.0) | 3.3 (1.2) |
| CRP (mg/L) | 10.3 (18) | 9.7 (16.1) | 8.4 (11.4) | 10.2 (17.6) | 11 (16.3) | 11.0 (20.4) | 14.5 (21.2) | 10.3 (17.6) | 9.7 (16.3) | 8.1 (14.4) | 11.1 (17.6) | 9.2 (15.2) |
| VAS general health patient (0-100) | 65.6 (25.5) | 46 (27) | 52 (24) | 56 (23) | 69.5 (26.1) | 51 (30) | 60 (25) | 62 (22) | 52.3 (29) | 37 (27) | 45 (23) | 44 (26) |
| HAQ score (0-3) | 0.8 (0.6) | 0.8 (0.6) | 0.6 (0.5) | 0.9 (0.6) | 1 (0.6) | 1.0 (0.7) | 0.8 (0.5) | 1.1 (0.6) | 0.6 (0.5) | 0.7 (0.6) | 0.5 (0.4) | 0.7 (0.6) |
| Swollen joint count (0-28), mean (sd) | 1.9 (2.8) | 2.3 (3.2) | 2.0 (3.1) | 3.1 (3.6) | 2.1 (3.3) | 1.7 (1.9) | 1.9 (3.7) | 2.7 (3.8) | 1.8 (3) | 1.5 (2.3) | 2.8 (3.2) | 2.3 (3.3) |
| Tender joint count (0-28), mean (sd) | 5.2 (6) | 3.2 (4.3) | 4.3 (5.2) | 5.3 (5.3) | 5.7 (6.4) | 3.2 (3.8) | 4.9 (6.5) | 5.8 (6.0) | 4 (5.2) | 2.2 (3.2) | 4.5 (4.4) | 3.5 (4.4) |
| **Concomitant treatments, (%)** |  |  |  |  |  |  |  |  |  |  |  |  |
| Any csDMARD | 874 (45.6) | 440 (74.5) | 549 (58.3) | 2751 (42.1) | 105 (25.3) | 71 (54.6) | 35 (39.3) | 445 (28.4) | 5712 (71.0) | 933 (60.6) | n/a | 5855 (62.9) |
| MTX | 664 (34.6) | 322 (54.5) | 579 (61.4) | 2412 (36.9) | 74 (17.8) | 51 (39.2) | 37 (43.5) | 392 (25.0) | 5002 (62.2) | 741 (48.1) | 161 (83.9) | 4999 (53.7) |
| Prednisolone | 156 (8.1) | 142 (24.0) | 245 (26.0) | 715 (10.9) | 49 (11.8) | 33 (25.4) | 18 (21.2) | 208 (13.3) | 739 (9.2) | 226 (14.7) | 126 (65.6) | 1138 (12.2) |
| **Comorbidities**, (%) |  |  |  |  |  |  |  |  |  |  |  |  |
| Cardiovascular disease | 111 (5.8) | 31 (5.2) | 51 (5.4) | 219 (3.3) | 26 (6.3) | 14 (10.8) | 8 (9.0) | 74 (4.7) | 444 (5.5) | 89 (5.8) | 10 (5.2) | 391 (4.2) |
| COPD | 26 (1.4) | 10 (1.7) | 13 (1.4) | 74 (1.1) | 14 (3.4) | 3 (2.3) | < 5 | 33 (2.1) | 139 (1.7) | 27 (1.8) | < 5 | 149 (1.6) |
| Diabetes mellitus | 100 (5.2) | 33 (5.6) | 66 (6.9) | 358 (5.5) | 25 (6.0) | 10 (7.7) | 9 (10.1) | 132 (8.4) | 368 (4.6) | 92 (6.0) | 10 (5.2) | 523 (5.6) |
| Hypertension | 141 (7.4) | n/a | n/a | 816 (12.5) | 47 (11.3) | n/a | n/a | 287 (18.3) | 625 (7.8) | n/a | n/a | 1282 (13.8) |
| Inflammatory bowel  disease | 51 (2.7) | n/a | n/a | 102 (1.6) | 8 (1.9) | n/a | n/a | 29 (1.9) | 112 (1.4) | n/a | n/a | 98 (1.1) |
| Number of hospitalizations at rheumatology /internal medicine department, mean (sd) | 0.7 (1.6) | n/a | 2.6 (4.0) | 0.4 (1.1) | 0.3 (1.0) | n/a | 0.7 (1.2) | 0.2 (0.8) | 1.7 (1.8) | n/a | 1.3 (2.0) | 0.2 (0.7) |
| **Dermatological comorbidity, (%)** |  |  |  |  |  |  |  |  |  |  |  |  |
| Cutaneous psoriasis | 643 (33.5) | 232 (39.3) | 324 (34.1) | 3790 (58.0) | 192 (46.3) | 72 (55.4) | 47 (52.8) | 1068 (68.2) | 2036 (25.3) | 575 (37.3) | 47 (24.5) | 4987 (53.5) |
| Actinic keratosis | < 3 | < 3 | 13 (1.4) | 77 (1.2) | < 3 | 0 (0.0) | < 5 | 25 (1.6) | 18 (0.2) | 9 (0.6) | < 5 | 138 (1.5) |
| Number of hospitalizations at dermatologic department, mean (sd) | 0.1 (0.8) | n/a | 0 (1) | 0 (0) | 0.1 (0.7) | n/a | < 5 | 0 (0) | 0.2 (1.1) | n/a | 0 (0) | 0 (0) |
| **Lifestyle factors** |  |  |  |  |  |  |  |  |  |  |  |  |
| Smoking |  |  |  |  |  |  |  |  |  |  |  |  |
| Current | 391 (20.4) | 86 (14.6) | 156 (16.4) | 405 (6.2) | 122 (29.4) | 28 (21.5) | 16 (18.0) | 152 (9.7) | 1064 (13.2) | 196 (12.7) | 35 (18.2) | 503 (5.4) |
| Former | 316 (16.5) | 39 (6.6) | 313 (32.9) | 1206 (18.4) | 85 (20.5) | 11 (8.5) | 23 (25.8) | 488 (31.2) | 1056 (13.1) | 95 (6.2) | 101 (52.6) | 1504 (16.1) |
| Never | 682 (35.6) | 338 (57.2) | 312 (32.8) | 1377 (21.1) | 163 (39.3) | 70 (53.8) | 18 (20.2) | 468 (29.9) | 2016 (25.1) | 1062 (69.0) | 52 (27.1) | 1484 (15.9) |
| Missing | 529 (27.6) | 128 (21.7) | 169 (17.8) | 3552 (54.3) | 45 (10.8) | 21 (16.2) | 32 (36.0) | 458 (29.2) | 3906 (48.6) | 187 (12.1) | < 5 | 5824 (62.5) |
| BMI, mean (SD) | 27.5 (5.4) | 29.1 (6.6) | n/a | n/a | 27 (5.5) | 29.9 (6.3) | n/a | n/a | 27.8 (5.5) | 28.7 (7.3) | n/a | n/a |
| Missing |  |  |  |  |  |  |  |  |  |  |  |  |

**Abbreviations**n/a: not available
PsA: Psoriatic Arthritis, bDMARD: biological disease modifying anti-rheumatic drug, csDMARD: conventional synthetic disease modifying anti-rheumatic drug, TNFi: Tumour Necrosis Factor inhibitor, non-TNFi bDMARD: encompasses abatacept, guselkumab, ixekizumab, kanakinumab, risankizumab, sekukinumab and ustekinumab, b/tsDMARD naïve: No exposure to a bDMARD or a targeted synthetic DMARD, CRR: Clinical Rheumatology Register, NPR: National Patient Registry, N: Number of, SD: Standard deviation, **,** IQR**:** interquartile range**,** CRP: C-reactive protein**,** VAS**:** visual analogue scale**,** HAQ**:** Health Assessment Questionnaire**,** DAS28-CRP: Disease Activity Score in 28 joints, BMI**:** body mass index**,** COPD: chronic obstructive pulmonary disease

**Supplementary Table S3.** Baseline characteristics for incident PsA patients from NPRs (Denmark and Sweden) and their age-, sex-, and calendar year-matched general population

| Cohort | PsA | PsA | GP | GP |
| --- | --- | --- | --- | --- |
| Country | dk | se | dk | se |
| N | 5125 | 18428 | 25873 | 92981 |
| Women, (%) | 2990 (58.3) | 9936 (53.9) | 15023 (58.1) | 50092 (53.9) |
| Age, years (SD) | 49.5 (14.4) | 52 (15) | 49.7 (14.5) | 52 (15) |
| **Country of birth, (%)** |  |  |  |  |
| Nordic | 4818 (94.0) | 17064 (92.6) | 21715 (83.9) | 77823 (83.7) |
| Non-Nordic | 307 (6.0) | 1364 (7.4) | 4158 (16.1) | 15158 (16.3) |
| **Educational level, (%)** |  |  |  |  |
| ≤9 years | 1160 (22.6) | 3180 (17.4) | 5378 (20.8) | 16013 (17.5) |
| 10-12 years | 2515 (49.1) | 5713 (31.2) | 11918 (46.1) | 24493 (26.7) |
| >12years | 1357 (26.5) | 9417 (51.4) | 7904 (30.5) | 51238 (55.8) |
| Calendar year (range) | 2010-2021 | 2010-2021 | 2010-2021 | 2010-2021 |
| **Concomitant treatments, (%)** |  |  |  |  |
| Any csDMARD | 1132 (22.1) | 8407 (45.6) | 107 (0) | 431 (0.5) |
| MTX | 958 (18.7) | 7024 (38.1) | 71 (0) | 163 (0.2) |
| Prednisolone | 435 (8.5) | 3750 (20.3) | 276 (0) | 887 (1.0) |
| **Comorbidities, (%)** |  |  |  |  |
| Cardiovascular disease | 335 (6.5) | 989 (5.4) | 1062 (4.1) | 3026 (3.3) |
| COPD | 131 (2.6) | 394 (2.1) | 310 (1.2) | 891 (1.0) |
| Diabetes mellitus | 243 (4.7) | 1275 (6.9) | 744 (2.9) | 3475 (3.7) |
| Hypertension | 455 (8.9) | 2859 (15.5) | 1271 (4.9) | 7355 (7.9) |
| Inflammatory bowel  disease | 96 (1.9) | 335 (1.8) | 202 (0.8) | 788 (0.8) |
| Number of hospitalizations at rheumatology /internal medicine department, mean (SD) | 1.1 (1.1) | 0.3 (1.0) | 0.1 (0.4) | 0.1 (0.7) |
| **Dermatological comorbidity at start of follow up, (%)** |  |  |  |  |
| Cutaneous psoriasis | 1936 (37.8) | 9911 (53.8) | 84 (0.3) | 1666 (1.8) |
| Actinic keratosis | 8 (0.2) | 276 (1.5) | 39 (0.2) | 1068 (1.1) |
| Number of hospitalizations at dermatologic department, mean (SD) | 0.4 (1.2) | 0.0 (0.3) | 0 (0.4) | 0.0 (0.0) |
|  |  |  |  |  |

**Abbreviations**
PsA: Psoriatic Arthritis, NPR: National Patient Registry, DK: Denmark, SE: Sweden, NO: Norway, FI: Finland, N: Number of, SD: Standard deviation, IQR: interquartile range, COPD: chronic obstructive pulmonary disease

**Supplementary Table S4.** Sensitivity analyses of PsA patient from CRR cohorts, alternate follow-up definitions. N events, person years, crude incidence rates and 95% confidence intervals for most recent drug + time-lagged most recent approaches by treatment cohort and by country (where N is at least 3).

| Country | Outcome | TNFi | | | non-TNFi bDMARD | | | b/tsDMARD naive | | |
| --- | --- | --- | --- | --- | --- | --- | --- | --- | --- | --- |
|  |  | N  events | PY | IR (95% CI) | N  events | PY | IR (95% CI) | N  events | PY | IR (95% CI) |
| **Most recent drug** |  |  |  |  |  |  |  |  |  |  |
| DK | NMSC | 35 | 9405 | 372.2 (259.2-517.6) | 4 | 1187 | 337.1 (91.8 - 863) | 219 | 48305 | 453.4 (395.3-517.6) |
| SE | NMSC | 95 | 22380 | 424.5 (347.2-519.1) | 15 | 3449 | 434.9 (262.2-721.4) | 196 | 38887 | 504 (438.2-579.7) |
| NO+FI | NMSC | 17 | 5821 | 292 (181.5-469.7) | < 3 | - | - | 17 | 6687 | 254.2 (158-408.9) |
|  |  |  |  |  |  |  |  |  |  |  |
| DK | BCC | 30 | 9414 | 318.6 (215-454.9) | < 3 | - | - | 184 | 48388 | 380.3 (327.3-439.3) |
| SE | BCC | 75 | 22405 | 334.7 (266.9-419.7) | 7 | 3453 | 202.7 (96.6-425.2) | 145 | 38958 | 372.2 (316.3-438.0) |
|  |  |  |  |  |  |  |  |  |  |  |
| DK | SCC | 7 | 9507 | 73.6 (29.6-151.7) | < 3 | - | - | 35 | 48956 | 71.5 (49.8-99.4) |
| SE | SCC | 6 | 22620 | 26.5 (11.9-59.0) | 3 | 3463 | 86.6 (27.9-268.5) | 24 | 39466 | 60.8 (40.8-90.7) |
| **Time-lagged most recent drug** |  |  |  |  |  |  |  |  |  |  |
| DK | NMSC | 35 | 9020 | 388 (270.3 - 539.6) | 5 | 1104 | 453.1 (147.1 - 1057.3) | 219 | 46323 | 472.8 (412.2-539.7) |
| SE | NMSC | 91 | 20791 | 437.7 (356.4-537.5) | 12 | 3073 | 390.5 (221.8-687.6) | 191 | 36727 | 520.1 (451.3-599.3) |
| NO+FI | NMSC | 16 | 5449 | 293.6 (179.9-479.2) | < 3 | - | - | 17 | 6268 | 271.2 (168.6-436.3) |
|  |  |  |  |  |  |  |  |  |  |  |
| DK | BCC | 30 | 9030 | 332.2 (224.1-474.3) | < 3 | - | - | 184 | 46406 | 396.5 (341.3-458.1) |
| SE | BCC | 73 | 20817 | 350.7 (278.8-441.1) | 6 | 3076 | 195 (87.6-434.1) | 143 | 36798 | 388.6 (329.9-457.8) |
|  |  |  |  |  |  |  |  |  |  |  |
| DK | SCC | 7 | 9124 | 76.7 (30.8-158.1) | < 3 | - | - | 35 | 46974 | 74.5 (51.9-103.6) |
| SE | SCC | 5 | 21032 | 23.8 (9.9-57.2) | 3 | 3086 | 97.2 (31.3-301.4) | 22 | 37306 | 59 (38.8-89.6) |

**Abbreviations**
PsA: Psoriatic Arthritis, NMSC: non-melanoma skin cancer; BCC: basal cell carcinoma; SCC: squamous cell carcinoma, DK: Denmark, SE: Sweden, NO: Norway, FI: Finland, bDMARD: biological disease modifying anti-rheumatic drug, TNFi: Tumour Necrosis Factor inhibitor, non-TNFi bDMARD: encompasses abatacept, guselkumab, ixekizumab, kanakinumab, risankizumab, sekukinumab and ustekinumab, b/tsDMARD naïve: No exposure to a bDMARD or a targeted synthetic DMARD, CRR: Clinical Rheumatology Register, N: Number of, PY: Person years, IR: Incidence rate per 100 000 PY.

**Supplementary Table S5.** Sensitivity analyses of PsA patient from CRR cohorts, alternate follow-up definitions. Hazard Ratios for all Cox models by country and pooled together by meta-analyses

| Analysis type | Outcome | bDMARD exposure | DK | DK | SE | SE | Meta-analysis |
| --- | --- | --- | --- | --- | --- | --- | --- |
|  |  |  | Cox model A, HR (95%CI) | Cox model B, HR (95%CI) | Cox model A, HR (95%CI) | Cox model B, HR (95%CI) | Cox model B, HR (95%CI) |
| Most recent drug |  | b/tsDMARD naive (ref.) |  |  |  |  |  |
|  | NMSC | TNFi | 1.04 (0.72-1.49) | 1.01 (0.68-1.5) | 1.21 (0.95-1.55) | 1.19 (0.93-1.52) | 1.15 (0.93-1.43) |
|  |  | Non-TNFi | 0.9 (0.33-2.45) | 0.74 (0.23-2.36) | 1.12 (0.66-1.89) | 1.07 (0.63-1.82) | 1.02 (0.62-1.67) |
|  | BCC | TNFi | 1.03 (0.7-1.51) | 0.92 (0.6-1.42) | 1.29 (0.97-1.73) | 1.30 (0.97-1.73) | 1.14 (0.82-1.57) |
|  |  | Non-TNFi | 0.53 (0.13-2.14) | 0.54 (0.13-2.22) | 0.74 (0.34-1.61) | 0.74 (0.34-1.61) | 0.69 (0.35-1.36) |
|  | SCC | TNFi | 1.48 (0.65-3.37) | 2.0 (0.85-4.71) | 0.83 (0.33-2.07) | 0.73 (0.29-1.85) | 1.23 (0.46-3.29) |
|  |  | Non-TNFi | 3.56 (0.81-15.61) | 2.82 (0.35-22.61) | 2.56 (0.71-9.21) | 2.03 (0.55-7.45) | 2.22 (0.74-6.70) |
|  |  |  |  |  |  |  |  |
| Time-lagged most recent drug |  | b/tsDMARD naïve (ref.) |  |  |  |  |  |
|  | NMSC | TNFi | 1.03 (0.72-1.47) | 1 (0.68-1.49) | 1.40 (1.09-1.79) | 1.40 (1.09-1.79) | 1.25 (0.87-1.78) |
|  |  | Non-TNFi | 1.1 (0.45-2.72) | 0.96 (0.35-2.66) | 0.99 (0.55-1.77) | 1.00 (0.56-1.78) | 1.01 (0.60-1.69) |
|  | BCC | TNFi | 1.02 (0.69-1.5) | 0.92 (0.6-1.41) | 1.52 (1.13-2.03) | 1.55 (1.15-2.07) | 1.22 (0.74-2.02) |
|  |  | Non-TNFi | 0.52 (0.13-2.11) | 0.53 (0.13-2.19) | 0.70 (0.30-1.61) | 0.73 (0.31-1.68) | 0.67 (0.32-1.38) |
|  | SCC | TNFi | 1.46 (0.64-3.32) | 1.98 (0.84-4.68) | 0.90 (0.33-2.43) | 0.83 (0.30-2.26) | 1.33 (0.57-3.14) |
|  |  | Non-TNFi | 5.04 (1.46-17.44) | 5.4 (1.15-25.36) | 2.94 (0.79-10.91) | 2.45 (0.65-9.26) | 3.43 (1.25-9.39) |

**Abbreviations**
PsA: Psoriatic Arthritis, NMSC: non-melanoma skin cancer; BCC: basal cell carcinoma; SCC: squamous cell carcinoma, DK: Denmark, SE: Sweden, bDMARD: biological disease modifying anti-rheumatic drug, TNFi: Tumour Necrosis Factor inhibitor, non-TNFi bDMARD: encompasses abatacept, guselkumab, ixekizumab, kanakinumab, risankizumab, sekukinumab and ustekinumab, b/tsDMARD naïve: No exposure to a bDMARD or a targeted synthetic DMARD, CRR: Clinical Rheumatology Register, HR: Hazard Ratio, 95%CI: 95% Confidence Intervals.
Cox model A: adjustment for sex and calendar period, and with attained age as the underlying time scale.
Cox model B: Cox Model A + adjustment for PsA disease duration, and with attained age as the underlying time scale.

**Supplementary Table S6.** Sensitivity analysis of PsA patient from CRR cohorts in the calendar period 2016 to 2021 only, main ‘ever-treated’ follow-up approach. N patients, N events, person years, crude incidence rates and 95% confidence intervals for Sweden and Denmark (where N is at least 3).

| Country | Outcome | TNFi | | | | non-TNFi bDMARD | | | | b/tsDMARD naive | | | |
| --- | --- | --- | --- | --- | --- | --- | --- | --- | --- | --- | --- | --- | --- |
|  |  | N patients | N  events | PY | IR (95% CI) | N patients | N  events | PY | IR (95% CI) | N patients | N  events | PY | IR (95% CI) |
|  |  |  |  |  |  |  |  |  |  |  |  |  |  |
| DK | NMSC | 1021 | X | X | 379.2 (189.3-678.4) | 371 | X | X | 481.4 (156.3-1123.5) | 3453 | 41 | 10456 | 392.1 (281.4-531.9) |
| SE | NMSC | 3793 | 52 | 11160 | 465.9 (355.0-611.4) | 1292 | 17 | 3896 | 436.3 (271.2-701.8) | 4290 | 47 | 9389 | 500.6 (376.1-666.3) |
|  |  |  |  |  |  |  |  |  |  |  |  |  |  |
| DK | BCC | 1021 | X | X | 309.7 (141.6-588) | 371 | X | X | 287.5 (59.3-840.3) | 3453 | 33 | 10467 | 315.3 (217-442.8) |
| SE | BCC | 3793 | 37 | 11176 | 331.1 (239.9-457) | 1292 | 9 | 3906 | 230.4 (119.9-442.8) | 4290 | 32 | 9402 | 340.4 (240.7-481.4) |
|  |  |  |  |  |  |  |  |  |  |  |  |  |  |
| DK | SCC | 1021 | < 3 | X | 68.4 (8.3-247.1) | 371 | < 3 | X | 192 (23.3-693.5) | 3453 | 7 | 10526 | 66.5 (26.7-137) |
| SE | SCC | 3793 | 4 | 11250 | 35.6 (13.4-94.9) | 1292 | 3 | 3915 | 76.6 (24.7-237.5) | 4290 | 4 | 9462 | 42.3 (15.9-112.7) |

**Abbreviations**
PsA: Psoriatic Arthritis, NMSC: non-melanoma skin cancer; BCC: basal cell carcinoma; SCC: squamous cell carcinoma, DK: Denmark, SE: Sweden, bDMARD: biological disease modifying anti-rheumatic drug, TNFi: Tumour Necrosis Factor inhibitor, non-TNFi bDMARD: encompasses abatacept, guselkumab, ixekizumab, kanakinumab, risankizumab, sekukinumab and ustekinumab, b/tsDMARD naïve: No exposure to a bDMARD or a targeted synthetic DMARD, CRR: Clinical Rheumatology Register, N: Number of, PY: Person years, IR: Incidence rate per 100 000 PY.
X: Indirect censoring due to Danish GDPR law and the possibility of backward calculations of N < 3 in this table or across the main analysis table.

**Supplementary Table S7.** Sensitivity analysis of PsA patient from CRR cohorts in the calendar period 2016 to 2021 only, main ‘ever-treated’ follow-up approach. Hazard ratios for all Cox models for Sweden and Denmark.

| Analysis type | Outcome | bDMARD exposure | DK | DK | SE | SE |
| --- | --- | --- | --- | --- | --- | --- |
|  |  |  | Cox model A,  HR (95%CI) | Cox model B,  HR (95%CI) | Cox model A, HR (95%CI) | Cox model B,  HR (95%CI) |
| Ever-treated approach |  | b/tsDMARD naive (ref.) |  |  |  |  |
|  | NMSC | TNFi | 1.2 (0.61-2.35) | 0.79 (0.34-1.84) | 1.28 (0.85-1.92) | 1.23 (0.81-1.85) |
|  |  | Non-TNFi | 1.7 (0.66-4.37) | 1.21 (0.40-3.63) | 1.07 (0.61-1.88) | 0.99 (0.56-1.75) |
|  | BCC | TNFi | 1.16 (0.55-2.45) | 0.6 (0.22-1.6) | 1.37 (0.84-2.24) | 1.32 (0.81-2.16) |
|  |  | Non-TNFi | 1.2 (0.36-3.96) | 0.61 (0.14-2.71) | 0.83 (0.39-1.75) | 0.77 (0.36-1.65) |
|  | SCC | TNFi | 1.46 (0.29-7.26) | 2.3 (0.38-14.15) | 1.37 (0.34-5.58) | 1.28 (0.31-5.31) |
|  |  | Non-TNFi | 4.97 (0.94-26.23) | 8.67 (1.23-60.96) | 2.48 (0.54-11.4) | 2.19 (0.45-10.6) |

**Abbreviations**
PsA: Psoriatic Arthritis, NMSC: non-melanoma skin cancer; BCC: basal cell carcinoma; SCC: squamous cell carcinoma, DK: Denmark, SE: Sweden, bDMARD: biological disease modifying anti-rheumatic drug, TNFi: Tumour Necrosis Factor inhibitor, non-TNFi bDMARD: encompasses abatacept, guselkumab, ixekizumab, kanakinumab, risankizumab, sekukinumab and ustekinumab, b/tsDMARD naïve: No exposure to a bDMARD or a targeted synthetic DMARD, CRR: Clinical Rheumatology Register, HR: Hazard Ratio, 95%CI: 95% Confidence Intervals.
Cox model A: adjustment for sex and calendar period, and with attained age as the underlying time scale.
Cox model B: Cox Model A + adjustment for PsA disease duration, and with attained age as the underlying time scale.

**Supplementary Table S8.** Sensitivity analyses of PsA patient from CRR cohorts, concomitant MTX use. Hazard Ratios for all Cox models by country and pooled together by meta-analyses

| Analysis type | Outcome | bDMARD exposure | DK | DK | SE | SE | Meta-analysis |
| --- | --- | --- | --- | --- | --- | --- | --- |
|  |  |  | Cox model A, HR (95%CI) | Cox model B, HR (95%CI) | Cox model A, HR (95%CI) | Cox model B, HR (95%CI) | Cox model B, HR (95%CI) |
| b/tsDMARD naive + MTX vs.  TNFi and non-TNFi bDMARDs |  | b/tsDMARD naive + MTX (ref.) |  |  |  |  |  |
|  | NMSC | TNFi | 1.2 (0.85-1.69) | 1.14 (0.78-1.67) | 1.21 (0.94-1.54) | 1.19 (0.93-1.53) | 1.18 (0.96-1.45) |
|  |  | Non-TNFi | 1.38 (0.63-2.99) | 0.97 (0.38-2.45) | 1.06 (0.68-1.66) | 1.03 (0.66-1.61) | 1.02 (0.68-1.52) |
|  | BCC | TNFi | 1.14 (0.78-1.67) | 1.04 (0.69-1.57) | 1.27 (0.96-1.69) | 1.28 (0.96-1.71) | 1.20 (0.94-1.52) |
|  |  | Non-TNFi | 0.7 (0.22-2.22) | 0.41 (0.1-1.72) | 0.93 (0.54-1.62) | 0.94 (0.54-1.65) | 0.82 (0.45-1.50) |
|  | SSC | TNFi | 1.7 (0.78-3.73) | 2.16 (0.9-5.19) | 1.10 (0.53-2.30) | 1.02 (0.49-2.14) | 1.43 (0.69-2.96) |
|  |  | Non-TNFi | 3.97 (1.12-14.08) | 4.5 (0.92-21.96) | 1.89 (0.58-6.10) | 1.67 (0.51-5.41) | 2.37 (0.92-6.11) |
|  |  |  |  |  |  |  |  |
| b/tsDMARD naive + MTX  vs.  TNFi + MTX and TNFi mono |  | b/tsDMARD naive + MTX (ref.) |  |  |  |  |  |
|  | NMSC | TNFi + MTX | 0.99 (0.58-1.69) | 1.08 (0.63-1.87) | 1.08 (0.81-1.45) | 1.07 (0.80-1.43) | 1.07 (0.83-1.39) |
|  |  | TNFi mono | 1.35 (0.9-2.04) | 1.19 (0.75-1.9) | 1.38 (1.02-1.86) | 1.37 (1.01-1.85) | 1.31 (1.02-1.69) |
|  | BCC | TNFi + MTX | 0.97 (0.54-1.72) | 1.01 (0.56-1.82) | 1.10 (0.79-1.54) | 1.11 (0.79-1.56) | 1.08 (0.81-1.46) |
|  |  | TNFi mono | 1.26 (0.8-1.98) | 1.06 (0.63-1.76) | 1.49 (1.06-2.09) | 1.51 (1.08-2.13) | 1.33 (0.95-1.88) |
|  | SSC | TNFi + MTX | 1.33 (0.4-4.47) | 1.82 (0.52-6.35) | 1.19 (0.51-2.76) | 1.08 (0.46-2.53) | 1.27 (0.63-2.57) |
|  |  | TNFi mono | 2.12 (0.85-5.3) | 2.55 (0.91-7.17) | 0.92 (0.33-2.55) | 0.89 (0.32-2.46) | 1.50 (0.53-4.23) |

**Abbreviations**
PsA: Psoriatic Arthritis, NMSC: non-melanoma skin cancer; BCC: basal cell carcinoma; SCC: squamous cell carcinoma, DK: Denmark, SE: Sweden, bDMARD: biological disease modifying anti-rheumatic drug, TNFi: Tumour Necrosis Factor inhibitor, non-TNFi bDMARD: encompasses abatacept, guselkumab, ixekizumab, kanakinumab, risankizumab, sekukinumab and ustekinumab, b/tsDMARD naïve: No exposure to a bDMARD or a targeted synthetic DMARD, CRR: Clinical Rheumatology Register, HR: Hazard Ratio, 95%CI: 95% Confidence Intervals, MTX: Methotrexate, mono: monotherapy at index, i.e., no concomitant MTX.
Cox model A: adjustment for sex and calendar period, and with attained age as the underlying time scale.
Cox model B: Cox Model A + adjustment for PsA disease duration, and with attained age as the underlying time scale.
